# Reconstruction of two-dimensional molecular structure with laser-induced electron diffraction from laser-aligned polyatomic molecules

**DOI:** 10.1038/srep15753

**Published:** 2015-10-27

**Authors:** Chao Yu, Hui Wei, Xu Wang, Anh-Thu Le, Ruifeng Lu, C. D. Lin

**Affiliations:** 1 Department of Applied Physics, Nanjing University of Science and Technology, Nanjing, Jiangsu 210094, PR China; 2 Department of Physics, Kansas State University, Manhattan, Kansas 66506, USA

## Abstract

Imaging the transient process of molecules has been a basic way to investigate photochemical reactions and dynamics. Based on laser-induced electron diffraction and partial one-dimensional molecular alignment, here we provide two effective methods for reconstructing two-dimensional structure of polyatomic molecules. We demonstrate that electron diffraction images in both scattering angles and broadband energy can be utilized to retrieve complementary structure information, including positions of light atoms. With picometre spatial resolution and the inherent femtosecond temporal resolution of lasers, laser-induced electron diffraction method offers significant opportunities for probing atomic motion in a large molecule in a typical pump-probe measurement.

Observation of isolated molecules undergoing structural transformations can provide the most direct insight into chemical processes. For static systems the majority of known molecular structures are determined using X-ray diffraction for crystallized molecules. For isolated molecules, conventional electron diffraction (CED) has been the main means for determining the bond distances of molecules in the gas phase but there has been no established method for measuring the three-dimensional (3D) structure^1^,^2^. To probe atomic motions in molecules or in condensed materials, in general temporal resolving power of a few to tens of femtoseconds is required. The recent development of femtosecond sources of free-electron X-ray lasers has been successfully demonstrated ultrafast X-ray diffraction for large biological molecules^3^ and nanocrystals^4^, but their present fluxes and energies are insufficient for ultrafast X-ray diffraction of isolated molecules^5^. Ultrafast electron diffraction (UED) has been used to probe changes in structure of molecules^6^,^7^,^8^ and extended materials^9^,^10^,^11^,^12^, but their time resolution has been limited to about one picosecond and often only the radial distances of the atoms are determined. While recent development of ultrafast electron diffraction has been used to probe dynamics in condensed medium^13^,^14^,^15^, the temporal resolution is still limited to a few hundred femtoseconds and has not been applied to isolated molecules. We note that ultrafast free-electron X-ray lasers and ultrafast electron diffraction employ either high-energy X-rays or high-energy electrons for achieving high spatial resolution, similar to the conventional X-ray or electron diffraction. The challenge is to develop femtosecond X-ray or electron pulses with enough flux for imaging.

Recently, an alternative laser-induced electron diffraction (LIED) method has been proposed for dynamic imaging of isolated molecules^16^. When molecules are exposed to an intense infrared laser pulse, near the field peak the molecule is ionized and the released electron is thrown into the oscillating laser field. As the field reverses its direction the electron may be driven back to recollide with its parent ion and elastically scatter off it. This last step is analogous to scattering of molecules by an external electron beam used in conventional electron diffraction (CED). If accurate structural information of the molecules can be reconstructed from such scattering images, then LIED can be used for dynamic imaging with few- to tens femtoseconds temporal resolutions since infrared lasers with such pulse durations are already available in many laboratories. However, there are several notable differences in LIED. First, in LIED the returning electrons are not monochromatic but broadband coherent electron pulses. Second, the kinetic energies of these electrons are far below the tens or hundreds of keV’s used in CED. Third, the scattering occurs in the presence of the laser field. The conditions for LIED to work were established in Xu *et al*^16^. It shows that LIED will work as CED if the returning electron energies are on the order of about 100–300 eV and if the diffraction images are taken at *backscattered* angles. For such collisions the momentum transfer in LIED has the same range as in CED where diffraction images are taken at forward directions (less than 20°). Under these conditions, LIED works exactly the same way as CED does, including the retrieval of molecular structure from the diffraction images, but with the added advantage of femtosecond temporal resolution of the laser pulses.

The first experimental demonstration of LIED was carried out for isotropically distributed N_2_ and O_2_ molecules with 2-micron lasers by Blaga *et al.*^17^. They retrieved N-N bond length in agreement with the known neutral N_2_ distance within the estimated resolution of 0.05 Å, but the O-O bond length was found to be 0.1 Å shorter than the neutral O_2_ bond length. The latter was attributed to the readjustment of O-O bond length within the 5 fs that takes the electron to return to collide with the molecular ion after tunnel ionization, thus providing the first evidence of sub-angstrom spatial and few-femtosecond temporal resolution with LIED. Other LIED experiments are emerging as more mid-infrared lasers become available. With 160 kHz, 3.1 μm lasers, Pullen *et al.*^18^ reported diffraction images of aligned and anti-aligned acetylene (C_2_H_2_) in coincidence with the molecular ions. The diffraction images were used to extract not only the C-C bond length but also the C-H bond length within the estimated resolution of 0.05 Å. In both cases, the bond distances were retrieved by *fitting* the experimental diffraction images from the photoelectron momentum spectra at fixed kinetic energy of the returning electrons using the Independent Atom Model (IAM)^1^. IAM is the standard model for CED and the bond distances are extracted by genetic algorithm fitting or by linear-square fitting. Since the returning electrons in LIED are broadband, the bond distances fitted from several fixed energies are compared for consistency.

To extend LIED beyond the proof-of-principle stage and apply it to more complex polyatomic molecules and to dynamic systems, the reconstruction of molecular structure with a fitting procedure clearly will become more difficult. Thus it is desirable to check if advanced retrieval methods employed by the X-ray^19^ and/or electron diffraction communities^20^ can be modified for LIED.

In this article, we extend the 2D Fourier transform method to retrieve 2D geometry from 1D-aligned symmetric-top polyatomic molecules using diffraction images obtained from LIED. Recall that LIED takes diffraction images at large scattering angles by *sub-keV* electrons. In the first method we modified the procedure used in UED by Hensley *et al.*^20^,^21^ where they extracted 2D molecular structure of CF_3_I from 1D *perpendicularly* aligned ensemble of molecules at a fixed electron energy. In the second method we took advantage of the broadband nature of the recollision wave packet, and proposed a new method to retrieve molecular structure from the 2D diffraction images in the (*E*,*θ*) plane, where *E* is the electron energy and *θ* is the scattering angle with respect to the incident electron beam (or polarization of the laser) after the signals have been integrated over the azimuthal angle. In this latter case, the molecules are aligned in 1D, *parallel to* the direction of the laser polarization. In the absence of experimental data, we generate “experimental data” using the IAM model and introduce 20% random errors to the data. These data are then used to retrieve the 2D molecular structure. As we will show below, the use of lower electron energies (sub-keV) and the broadband nature of the recolliding electrons allow determination of some atoms that are normally invisible in X-ray diffraction or in CED. Both features are important advantages of LIED since a single LIED measurement amounts to taking multiple diffraction images. These different images can be reconstructed to provide complementary molecular structure information for a more complete understanding of the system under study. Ideally 3D structure of an asymmetric polyatomic molecule may be retrieved if molecules are 3D aligned and such an investigation has been proposed recently^22^. However, 3D alignment of molecules in general is much more difficult^23^,^24^ and we do not expect LIED experiments on 3D aligned molecules to appear soon. We comment that Xu *et al.*^25^ has recently employed 1D Fourier transform method to reconstruct bond length using LIED data from nonaligned N_2_ molecules.

## Results

### Retrieval of structure from perfectly 1D aligned molecules at a fixed collision energy

**Figure 1:**
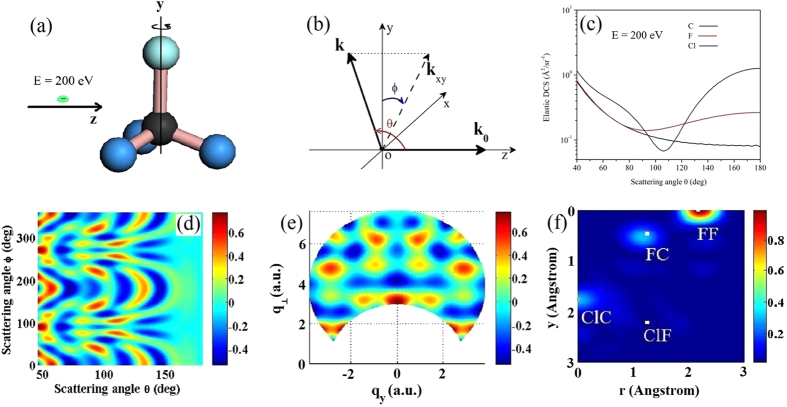
Reconstruction of 2D molecular structure by LIED from perfectly 1D aligned ensemble of ClCF_3_ molecules. (**a**) The returning electron beam is taken at 200 eV and along the z-axis, while the Cl-C axis lies along the y-axis. The whole molecule is freely rotating about the y-axis. (**b**) Geometry of incident and scattered electron momentum vectors and angles. (**c**) Elastic scattering differential cross sections (DCS) of C^+^, F^+^ and Cl^+^ ions for electron impact at 200 eV. (**d**) Simulated molecular contrast factors (MCF) against the scattering angles *θ* and *φ*. (**e**) The same MCF expressed in terms of 2D momentum transfer components q_y_ and q_⊥_. The white areas correspond to regions that were excluded from consideration for molecular structure retrieval since DCS at angles less than 45° were excluded for diffraction consideration. (**f**) 2D molecular structure retrieved from the diffraction images shown in (**e**). The Cl is located at the origin (upper left corner). Each bright point indicates the projection of the bond length vector along (y) and perpendicular (r) to the alignment axis Cl-C, respectively. The retrieved bond lengths agree with the input data except that the Cl-F bond length is not visible.

Figure 1(a) depicts the geometry of a conventional electron diffraction experiment from an ensemble of perfectly 1D aligned molecules, here taken to be CF_3_Cl. The incident electron is taking to be along the z-axis and the molecules are aligned along the y-axis, which is along the Cl-C pair. The incident momentum 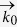 and the scattered momentum 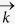 in the chosen coordinate frame are shown in Fig. 1(b). To simulate experiments using LIED, we took the incident electron energy at 200 eV, and the electron scattering images are taken at large angles [45° to 180°, see Fig. 1(d)]. This is different from the standard electron diffraction where the collision energy is about 20 keV and the scattering angles are typically between 0° and 20°. In the absence of experimental data, we assume that elastic scattering differential cross sections (DCS) can be calculated from IAM^1^,^16^. In IAM, a molecule is regarded as a collection of atoms fixed in space. If atom *i* is fixed at 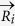 and *f*_*i*_ is the complex scattering amplitude by the incident electron, the total scattering intensity is


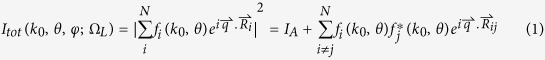


Here *N* is the total number of atoms in the molecule, 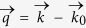 is the momentum transfer between the incident momentum 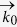 and the scattered momentum 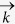, *q* = 2 *k*_0_ sin(*θ*/2), *Ω*_*L*_ is the molecular alignment angle, *θ* and *φ* are the electron scattering angles, and 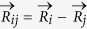. The first term on the right-hand side, 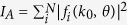, is an incoherent sum of scattering cross sections from all the atoms in the molecule. It carries no molecular structure information. That information is contained only in the second term which is called the molecular interference term. We define a 2D molecular contrast factor (MCF): 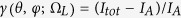, which is the ratio of the oscillatory molecular term to the smooth atomic term. Clearly oscillation of the MCF will yield the structural parameters 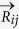 of the molecule. Figure 1(d) shows the 2D MCF pattern for perfectly 1D vertically aligned ClCF_3_ molecules. In the calculation the molecules are free to spin about the molecular axis. Thus the MCF is incoherently averaged over this rotation. Since the molecules are only 1D aligned, each orientation in Fig. 1(a) is accompanied by a center-inverted one. We set bond lengths *r*_*Cl-C*_ =1.751 Ǻ, *r*_*C-F*_ =1.342 Ǻ, and bond angle ∠F-C-F = 108.6° for ClCF_3_. Backscattering electrons are used in this paper, so we only keep the range of MCF with scattering angle *θ* > 45°.

For structure reconstruction, first we express the MCF in terms of momentum transfer along the alignment axis, q_y_, and its perpendicular component, q_⊥_ (The transformation is detailed in the Supplementary Information.) The results are shown in Fig. 1(e). (Only positive q_⊥_ is shown due to symmetry.) Due to the constraint that *θ* is larger than 45°, diffraction images from small q_⊥_ components are not used. By Fourier transforming the 2D MCF from momentum space to real space 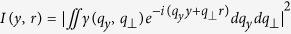, 2D molecular structure information can be obtained in cylindrical coordinates (*y*, *r*), as shown in Fig. 1(f). In this figure, the Cl atom is at the origin (upper-left corner). The expected input 2D bond lengths (projected with respect to the alignment axis) are marked by bright white dots. The Cl-C, F-C and F-F bond lengths are clearly retrieved and in good agreement with the distances marked by the white dots. On the other hand, the Cl-F bond length is not clearly retrieved. For comparison, in standard high-energy (25 keV) electron diffraction from 1D aligned CF_3_I molecules, Hensley *et al.*^20^ were able to retrieve only the I-C and I-F bond lengths (also the ∠I-C-F bond angle). The bond lengths C-F and F-F are not retrieved in their experiment.

The difference between the two results exemplifies the advantage of using “low-energy” (a few-hundred eV’s) electrons for taking diffraction images. With tens of keV electrons, the elastic scattering differential cross sections (DCS) are always forwardly peaked where the scattering amplitude is approximately proportional to the nuclear charge. With high-energy electron diffraction, the incident electron is not scattered by light atoms, thus no information about the low-Z atoms can be retrieved. With ‘low-energy” electron diffraction, the diffraction images are taken at large scattering angles, the same range of momentum transfer q can be reached as in high-energy electron diffraction. These high-*q* core-penetrating hard collisions reveal the atomic cores in the molecule. For “low-energy” large angle scattering, the DCS often exhibits oscillatory structures for heavy atoms. In Fig. 1(c), we show the DCS for electron collisions with C^+^, F^+^, and Cl^+^ (practically identical to their respective neutral target) at 200 eV. For Cl, there is a sharp minimum near 110°. For scattering angles near this minimum, the Cl is not visible even though it is the heaviest atom. Since the DCS for Cl in the few-hundred eV’s range changes rapidly with energy, by varying the collision energy, we can expect Cl to become visible at different collision energies (see below).

### Retrieval of structure from partially 1D aligned molecules at a fixed collision energy

**Figure 2:**
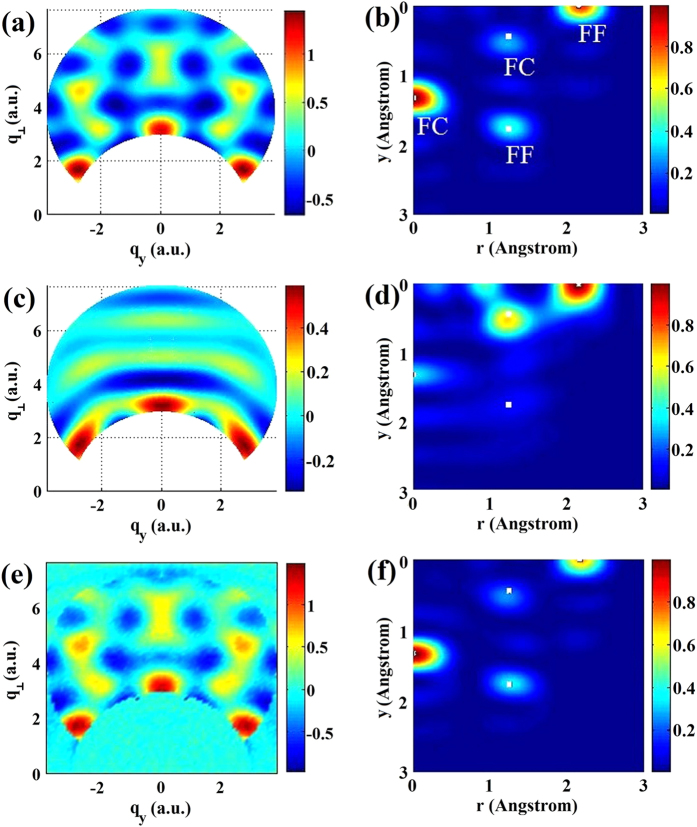
2D diffraction images and reconstructed 2D molecular structures for CF_4_. (**a,b**) For perfectly 1D perpendicularly aligned molecules; (**c,d**) For partially 1D perpendicularly aligned molecules. (**e,f**) For perfectly 1D aligned molecules iteratively extracted from partially aligned ones shown in (**c**).

Experimentally molecules cannot be perfectly 1D aligned, but partially aligned with the molecular axis precessing about the alignment axis. Assume that the alignment distribution has the Gaussian form 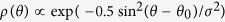, where *θ*_*0*_ =π/2 corresponds to molecules being vertically aligned. If the molecules, taken to be CF_4_, are perfectly 1D aligned, then from the IAM model, the calculated MCF *γ*(*q*_y_, *q*_⊥_) and the retrieved 2D molecular structure using the same method as in Fig. 1, are shown in Fig. 2(a,b). In this case, all the bond lengths (as well as the bond angle ∠F-C-F) can be retrieved. If we use the MCF calculated for the partially aligned CF_4_ molecules, taken σ^2^ = 0.04, the resulting *γ*(*q*_y_, *q*_⊥_), shown in Fig. 2(c), begins to lose the sharp diffraction peaks, as compared to the perfectly aligned one shown in Fig. 2(a). Using the 2D Fourier transform, the retrieved 2D molecular structure, shown in Fig. 2(d), becomes seriously degraded and unphysical peaks begin to appear. Such retrieved results are not acceptable.

A better procedure to retrieve 2D molecular structure from partially 1D aligned molecules is to first extract perfectly aligned MCF from the partially aligned MCF, using the known alignment angular distribution, by an iterative method. Figure 2(e) shows the retrieved MCF for perfectly 1D aligned one from the partially aligned MCF shown in Fig. 2(c). Comparing to Fig. 2(a) for the MCF calculated for perfectly 1D aligned molecules, we can see that this iteration method was able to duplicate Fig. 2(a) very well. Using the 2D Fourier transform the retrieved 2D molecular structure, shown in Fig. 2(f), is in good agreement with Fig. 2(b).

Briefly, the iteration method is carried out as follows. First, we guess an MCF pattern for molecules that are perfectly aligned. From this initial guess, the MCF for molecules at another fixed aligned angle is calculated using a formula given in the Supplementary Information. Second, with the known angular distribution, the MCF for the partially aligned molecules is obtained. This intermediate MCF is compared to the experimental data. In the third step, a small random distribution is added to the initial guess of the perfectly aligned molecules, from which a new MCF for the same alignment distribution is calculated and then compared to the experimental data. Only if the change reduces the error will the new MCF distribution be kept. This process is repeated over about 50k iterations. To eliminate randomness, we averaged 20 independent iteration results. The Supplementary Information gives additional details of this procedure.

### Retrieval of structure from partially 1D aligned molecules with broadband recollision energies

In LIED experiments, the rescattering electron is represented by a broadband wave packet. Recently, this energy dimension has also been shown^25^ to be an effective way for structural retrieval at a fixed scattering angle θ = 180°. Here we put forward a second method of new 2D structural retrieval using 2D diffraction image (*p,θ*) where *p* is the momentum value of the electron and *θ* is the scattering angle with respect to the incident beam direction, obtained by integrating over the azimuthal angle *φ*. In this method, the molecules are aligned along the direction of the incident electrons [See Supplementary Fig. S3]. In the simulation below, the electron energy is taken in the range of 100 eV < E < 300 eV where the DCS is accurately described by the IAM. The 2D MCF obtained is calculated from


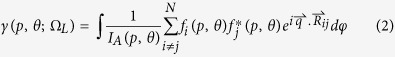


where *p* is the momentum of the incident electrons, and 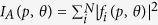 is the total atomic DCS. In this model, molecules are 1D parallel aligned along the z-axis.

**Figure 3:**
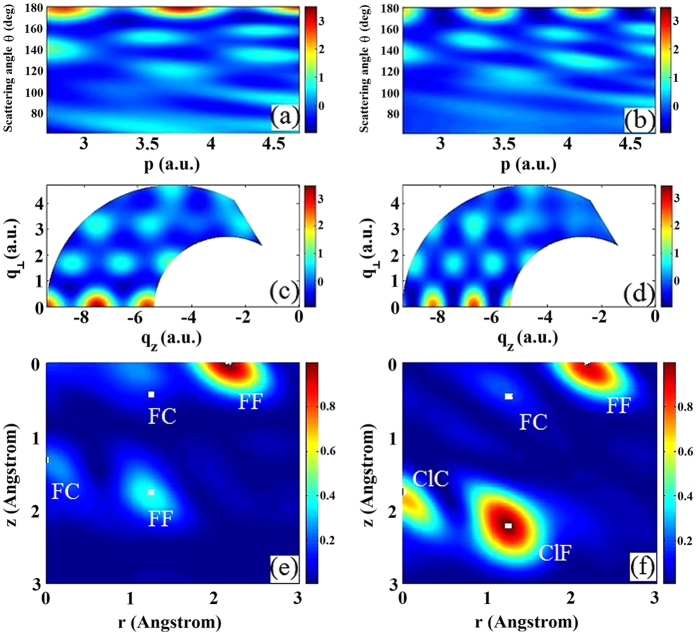
2D diffraction images and reconstructed 2D molecular structures from perfectly 1D aligned CF_4_ (left column) and ClCF_3_ (right column). The molecules are aligned parallel to the direction of the broadband (100–300 eV) electron beam. (**a,b**) Diffraction images in polar coordinates; (**c,d**) Diffraction images in two-component momentum transfer coordinates; (**e,f**) 2D Fourier transformed molecular structures.

Figure 3(a,b) show the 2D MCF, *γ*(*p*,*θ*), for perfectly 1D aligned CF_4_ and ClCF_3_, respectively. We keep the range of MCF to scattering angle *θ* > 60° only. In Fig. 3(c,d), the MCF is expressed in (*q*_z_, *q*_⊥_) using the relation: 
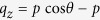
 and 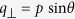. Here *q*_z_ is parallel to the molecular axis, and *q*_⊥_ perpendicular to it. The 2D Fourier transform is then used to obtain the 2D molecular structure of CF_4_ and ClCF_3_, shown in Fig. 3(e,f), respectively. Unlike the first method, the most effective angular region is found to be between 120° < *θ* < 180° in this method. Thus the F-F peak is stronger than the weak F-C peak in Fig 3(e) for CF_4_. For ClCF_3_ the Cl-F peak is stronger than the F-C peak. At angles larger than 120°, Fig. 1(c) [or Supplementary Fig. S3] shows that the DCS decreases in the order of Cl, F and C. Thus C is more difficult to be observed for the present cases with this method. The result illustrates our assertion that bond lengths extracted at different collision energy and/or different scattering angles can provide complimentary structure information. Some bond lengths that are not easily visible using method 1 can be more clearly seen using method 2, and vice versa.

The present method can be applied to molecules that are partially 1D aligned, using the same iterative procedure as before. Our simulation shows that with the present method, the diffraction images depend less sensitively on the alignment angles, and thus accurate 2D molecular structure can be retrieved even if the alignment is not very good. Additional examples can be seen in the Supplementary Information.

### “Molecular Movies”

**Figure 4:**
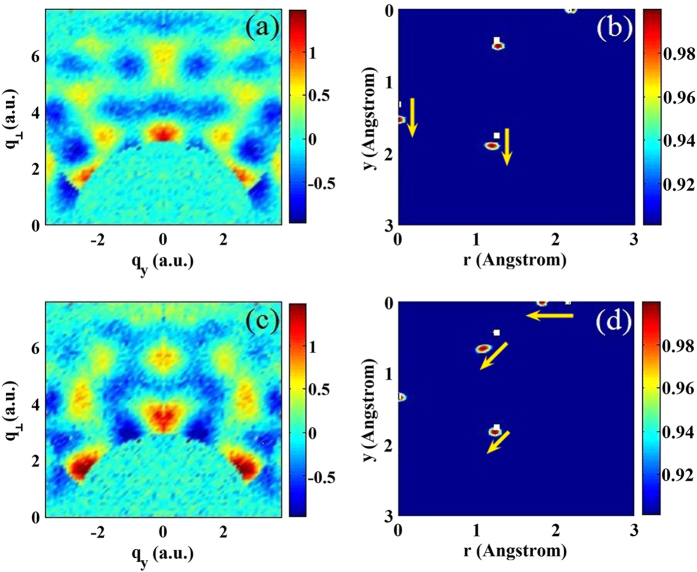
Extracted diffraction images from partially 1D aligned molecules and the reconstructed 2D molecular structures. The white dots are bond length coordinates for the equilibrium geometry of CF_4_ and the retrieved data are for: (**a,b**) the C-F distance along the alignment axis being extended from 1.323 Å to 1.60 Å; (**c,d**) the bond angles ∠F-C-F from the F atom along the alignment axis being extended from the original 109.5° to 124.5°.

The major motivation of LIED is to probe the motion of atoms in a molecule, i.e., to make a molecular movie in their own timescales. In Fig. 4(a,b), we simulate how the diffraction images and the retrieved 2D molecular structures are changed if the fluorine atom of CF_4_ along the alignment axis is displaced from 1.323 Ǻ to 1.60 Ǻ, while keeping all the other atoms unchanged. Similarly, Fig. 4(c,d) show the results when we change the three bond angles ∠F-C-F from 109.5° to 124.5°. (The first F in the bond angles refers to the F atom on the alignment axis.) These simulations illustrate how a molecular “movie” can be constructed using LIED. As the motions of atoms in a molecule evolve in time, the sequence of diffraction images, by a 2D Fourier transform, can be converted to a sequence of pictures giving new positions of atoms in the molecule. Playing out these pictures sequentially gives a direct visualization of the motion of atoms in time, i.e., a true “molecular movie” in the conventional coordinate space.

## Discussion

Comparing to the reported LIED results^17^,^18^ for N_2_, O_2_, and C_2_H_2_, here we have demonstrated that LIED-based imaging approach can be extended to larger gas-phase polyatomic molecules. When molecules are 1D aligned, 2D molecular structure information can be readily retrieved using the 2D Fourier transform. Today few-tens femtosecond mid-infrared lasers are already available, so the 2D retrieval methods presented here would allow the reconstruction of a “molecular movie” with few-tens femtosecond temporal and sub-angstrom spatial resolutions in a pump-probe type experiment. 1D nonadiabatic alignment of polyatomic molecules has been carried out in many strong field laboratories worldwide. Thus there is no obvious reason that LIED experiments simulated here cannot be carried out with the present-day technology. Looking ahead, the flux of the returning electrons (or the yield of the diffraction images) can be enhanced by several orders of magnitude with the emerging laser technologies, by increasing the repetition rate of typical kHz lasers to hundreds kHz or even MHz^26^,^27^. The returning electron flux can also be substantially enhanced by using synthesized two-color or three-color pulses^28^,^29^. In addition, the diffracted electrons can also be measured in coincidence with fragmented molecular ions^18^ to reduce background electron noises. Clearly there are ample opportunities for LIED to emerge as a powerful table-top tool for imaging molecular dynamics.

There are several new technologies under development aiming at ultrafast dynamic imaging of isolated molecules. High-flux free-electron X-ray lasers have been used to image 2,5-diiodobenzonitrile using 2-keV radiation from the LCLS X-ray laser^5^ and bond length between the two iodine atoms has been retrieved to be at 800 pm, as compared to the known value of 700 pm. The cross section for X-ray diffraction is six orders of magnitude smaller than that for electron diffraction, thus in general electron diffraction is preferable for gas-phase molecule imaging. For ultrafast electron diffraction currently available, few-tens keV electron pulses are limited to a few hundred femtoseconds. Other time-resolved electron diffraction methods include inner-shell photoelectron diffraction (ISPD)^30^,^31^ and laser-assisted electron diffraction (LAED)^32^. The latter is electron diffraction in the presence of an infrared laser. The time resolution is provided by the X-ray lasers for ISPD and by the pulse duration of the driving IR laser for LAED. All of these methods employ high-energy X-rays or electrons. They are unable to probe light atoms in the molecules. Compared to LIED, these methods use monochromatic high-energy electrons or photons, and thus would achieve higher spatial resolution. In comparison, in LIED the spatial resolution is set to be about 5 pm when used with the broadband sub-keV electrons. Such spatial resolution is adequate for imaging dynamics of a molecule under conformal transformation.

## Additional Information

**How to cite this article**: Yu, C. *et al.* Reconstruction of two-dimensional molecular structure with laser-induced electron diffraction from laser-aligned polyatomic molecules. *Sci. Rep.* **5**, 15753; doi: 10.1038/srep15753 (2015).

## Supplementary Material

Supplementary Information
