# Supplementary material for: Reconstruction of two-dimensional molecular structure with laser-induced electron diffraction from laser-aligned polyatomic molecules
Source: Sci Rep. 2015 Oct 27;5:15753. doi: 10.1038/srep15753 (PMC4621501; doi:10.1038/srep15753)
Supplement: Supplementary Information [file srep15753-s1.pdf]

# Supplementary Information for

## Reconstruction of two-dimensional molecular structure with laser-induced electron diffraction from laser-aligned polyatomic molecules

Chao Yu[1,2], Hui Wei[2], Xu Wang[2], Anh-Thu Le[2], Ruifeng Lu[1,*] & C. D. Lin[2,*]

[1] Department of Applied Physics, Nanjing University of Science and Technology, Nanjing, Jiangsu 210094, PR China

[2] Department of Physics, Kansas State University, Manhattan, Kansas 66506, USA

## Supplementary Methods

### Transformation of angle-dependent molecular contrast factor (MCF) to momentum transfer plane

The diffraction images expressed as molecular contrast factor in scattering angles ($\theta, \varphi$) is converted to the momentum transfer plane ($q_y$, $q_\perp$). From Fig. 1(b),

$$q_x = |\vec{q}|\sin(\frac{\pi-\theta}{2})\sin(\varphi) = k_0\sin(\theta)\sin(\varphi),$$
$$q_z = -|\vec{q}|\cos(\frac{\pi-\theta}{2}) = -2k_0\sin^2(\frac{\theta}{2}),$$
$$q_y = |\vec{q}|\sin(\frac{\pi-\theta}{2})\cos(\varphi) = k_0\sin(\theta)\cos(\varphi),$$
$$q_\perp = \sqrt{q_x^2 + q_z^2}$$

Once $\gamma(q_y, q_\perp)$ is obtained, a 2D Fourier transform is carried out to obtain the 2D molecular structure represented by the distribution:

$$I(y,r) = \left|\iint \gamma(q_y, q_\perp) e^{-i(q_y y + q_\perp r)} dq_y dq_\perp\right|^2$$

**Iterative method to retrieve MCF for perfectly 1D aligned molecules from partially 1D aligned molecules**

In Fig. S1 we illustrate the limitations of retrieving molecular structures directly from partially 1D perpendicularly aligned molecules. We consider the alignment distribution to be given in the following Gaussian form

$$\rho(\theta) \propto \exp(-0.5\sin^2(\theta-\theta_0)/\sigma^2),$$

where $\theta_0 = \pi/2$ corresponds to molecules being vertically aligned. Fig. S1(b) shows three distributions with $\sigma^2 = 0.02$, 0.04 and 0.08. The MCF extracted from "experimental" diffraction images expressed on ($q_y$, $q_\perp$) are displayed in Figs. S1 (c)-(e), and the reconstructed 2D molecular structures are shown in Figs. S1 (f)-(h), for the three alignment distributions, respectively. Clearly, the directly retrieved results are not acceptable. They are much inferior compared to Fig. 2(f) in the main text.

The iterative method is based on the principle that two different MCF patterns, $\gamma_A(\theta, \varphi)$ and $\gamma_B(\theta, \varphi)$, are related to each other. Here *A* refers to molecules that are perpendicularly aligned with respect to the electron beam, while the alignment axis of *B* has angles ($\theta_0$, $\varphi_0$) with respect to *A*. Since the DCS depends on the momentum transfer only, the two points $\gamma_A(\theta, \varphi)$ and $\gamma_B(\theta, \varphi)$ will have the same MCF if they have the same momentum transfer, i.e., the same $q_\parallel$ and $q_\perp$. The two sets of $q_\parallel$ and $q_\perp$ are related by

$$\begin{cases} q_{Ay} = (q_{Bx}, q_{By}, q_{Bz})\cdot(\sin\theta_0\sin\varphi_0, \sin\theta_0\cos\varphi_0, \cos\theta_0) \\ q_{Ay}{}^2 + q_{A\perp}{}^2 = q_{Bx}{}^2 + q_{By}{}^2 + q_{Bz}{}^2 \end{cases},$$

where the first equation expresses the equality for the component parallel to the molecular axis. This relation allows us to find $\gamma_B(\theta, \varphi)$ from $\gamma_A(\theta, \varphi)$. A four-point interpolation scheme was used to obtain $\gamma_B(\theta, \varphi)$ from $\gamma_A(\theta, \varphi)$.

To test the accuracy of this method, we compare the MCF calculated directly from the IAM, for 1D perfectly aligned molecules for molecular axis at $\theta_0$=80°, 70° and 60°, respectively, on the top row of Fig. S2, with the MCF obtained by transforming from the MCF at $\theta_0$=90° on the bottom row of Fig. S2. Outside of the missing region at large angles the two sets of data agree quite well. Since the missing region increases as the alignment angle $\theta_0$ deviates more from the vertical direction, the result indicates that the method is effective only if the molecules have a high degree of 1D alignment.

For the initial guess of the MCF for perfectly aligned molecules in the iteration method, we may start with a uniform distribution on the 2D space. This will lead to slower convergence. One may also use the IAM model to obtain the initial guess for MCF with

some approximate bond lengths. This works well for static molecules. For dynamic systems, the initial guess can always depend on the retrieved results from the prior time step.

**2D retrieval of molecular structure from partially 1D parallel aligned molecules with broadband electron recollision energies**

In this method, the molecular axis is partially parallel aligned with respect to the direction of the incident electrons (or laser polarization axis), as indicated in Fig. S4(a). Three examples of alignment distributions are shown in Fig. S4(b). Figs. S4(c)-(h) demonstrate that the 1D perfectly aligned MCF retrieved from these different alignment distributions (the left column for $CF_4$ and the right column for $ClCF_3$) give nearly identical diffraction patterns, illustrating that the retrieval is less sensitive to alignment distributions. This conclusion is also confirmed by the retrieved 2D molecular structures shown in Fig. S5, where the results from different alignment distributions are essentially the same.

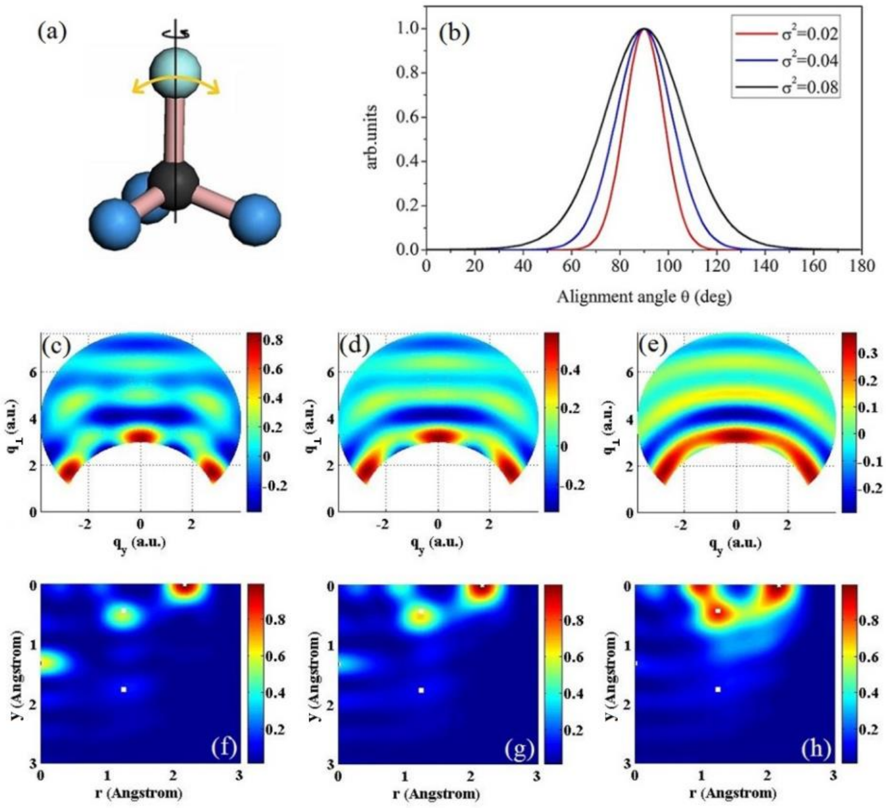


Supplementary Figure 1: For partially 1D aligned $CF_4$ molecules. (a) Partial 1D alignment of molecules. The inverted arrangement is not shown. (b) Three partial 1D perpendicular alignment distributions. (c-e) Extracted diffraction images (or more precisely, MCF) for the three partial alignment distributions. (f-h) 2D molecular structures obtained from the diffraction images directly using 2D Fourier transform. The results show that partial alignment destroys accurate retrievable 2D molecular structures.

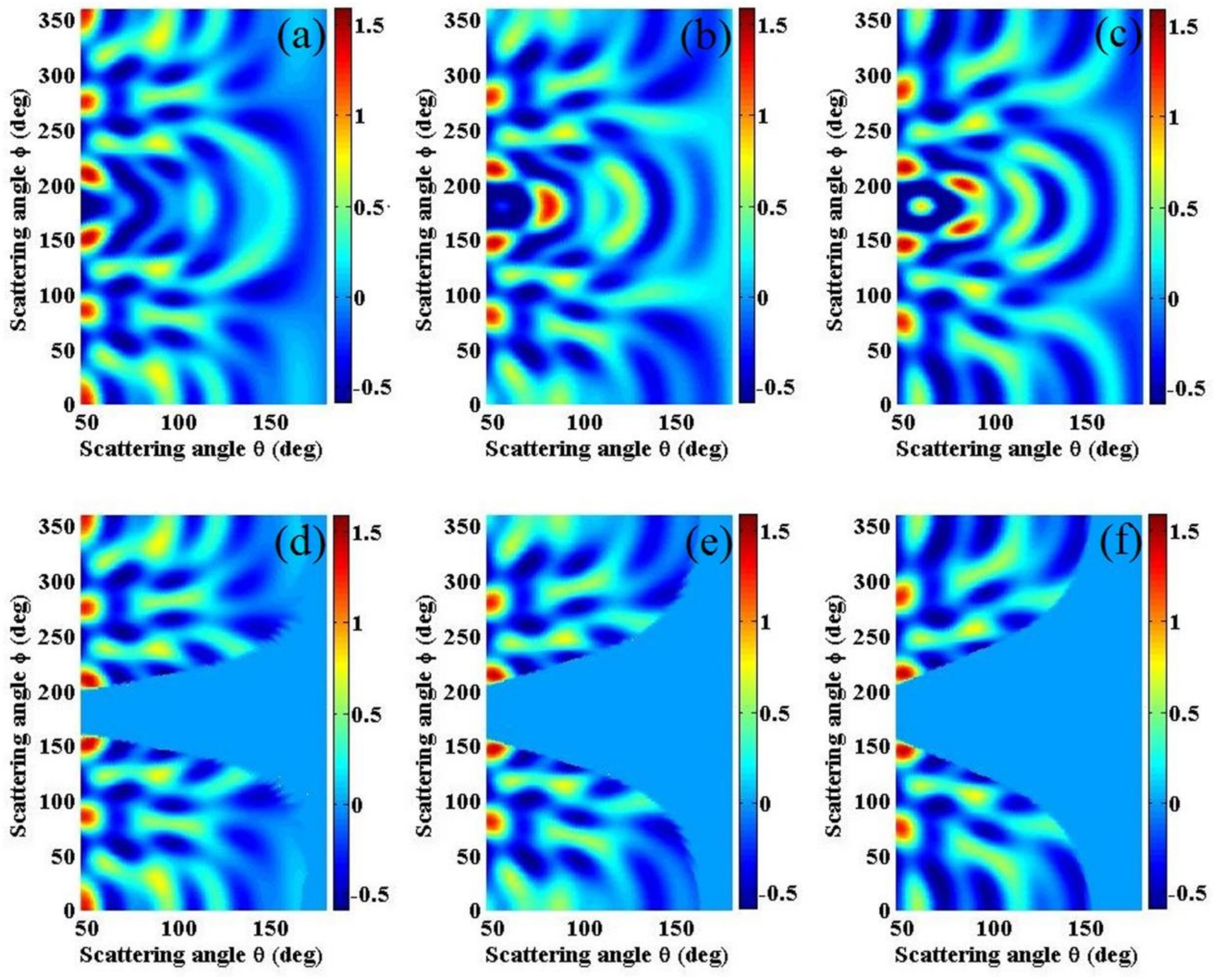


Supplementary Figure 2: (a-c) MCF calculated directly using the IAM model for molecules aligned at 80$^o$, 70$^o$ and 60$^o$ with respect to the vertical alignment axis for $CF_4$, respectively. (d-f) The MCF for aligned angles of 80$^o$, 70$^o$ and 60$^o$, obtained from MCF at 90$^o$ using the method described in the Supplementary Methods. The empty region in (d-f) corresponds to angular ranges not accessible in the transformation.

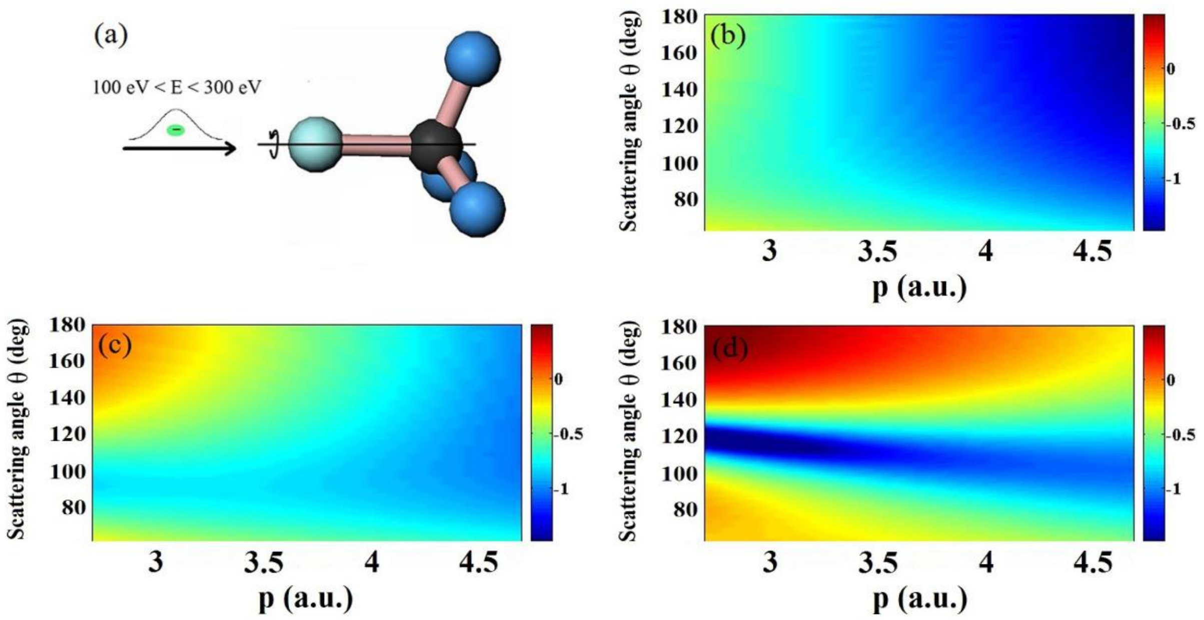


Supplementary Figure 3: (a) Schematic illustration for broadband electron diffraction from 1D perfectly parallel aligned molecules. (b-d) Elastic differential scattering cross section (DCS) above $60^{o}$ for 100-300 eV electrons, for (b) carbon, (c) fluorine, and (d) chlorine. Minima in DCS can be seen in F and in Cl. The DCS for C decreases monotonically in the covered range. Note in the considered angular and momentum ranges the DCS are the same for neutral atom and singly charged ion.

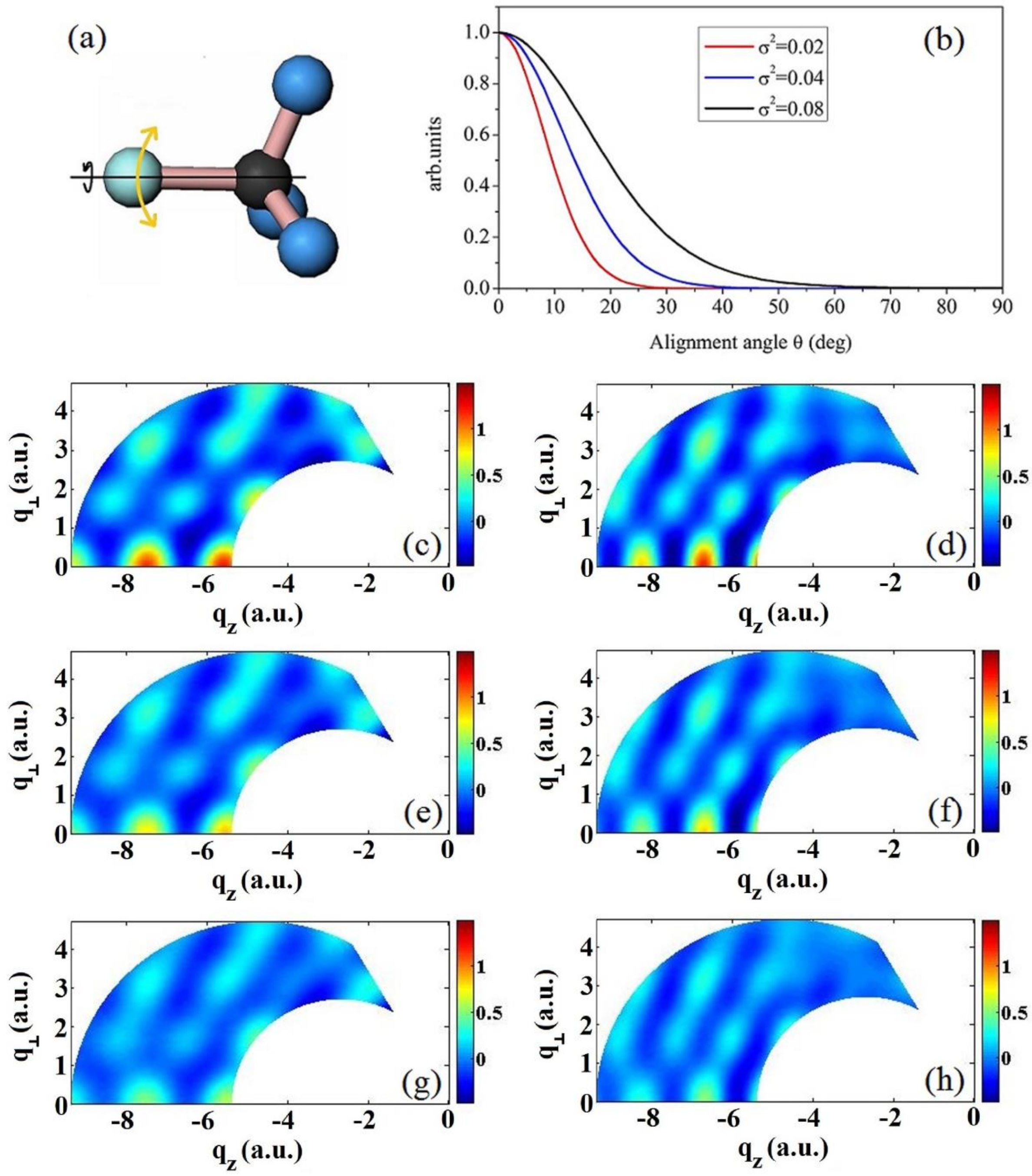


Supplementary Figure 4: (a) Geometry for diffraction with a broadband electron beam from partially 1D parallel aligned molecules. (b) Illustration of three different alignment distributions. (c,e,g) MCF for $CF_4$ iteratively extracted for perfectly 1D aligned ensemble starting with the three alignment distributions. (d,f,h) The same as the left three panels, but for $ClCF_3$.

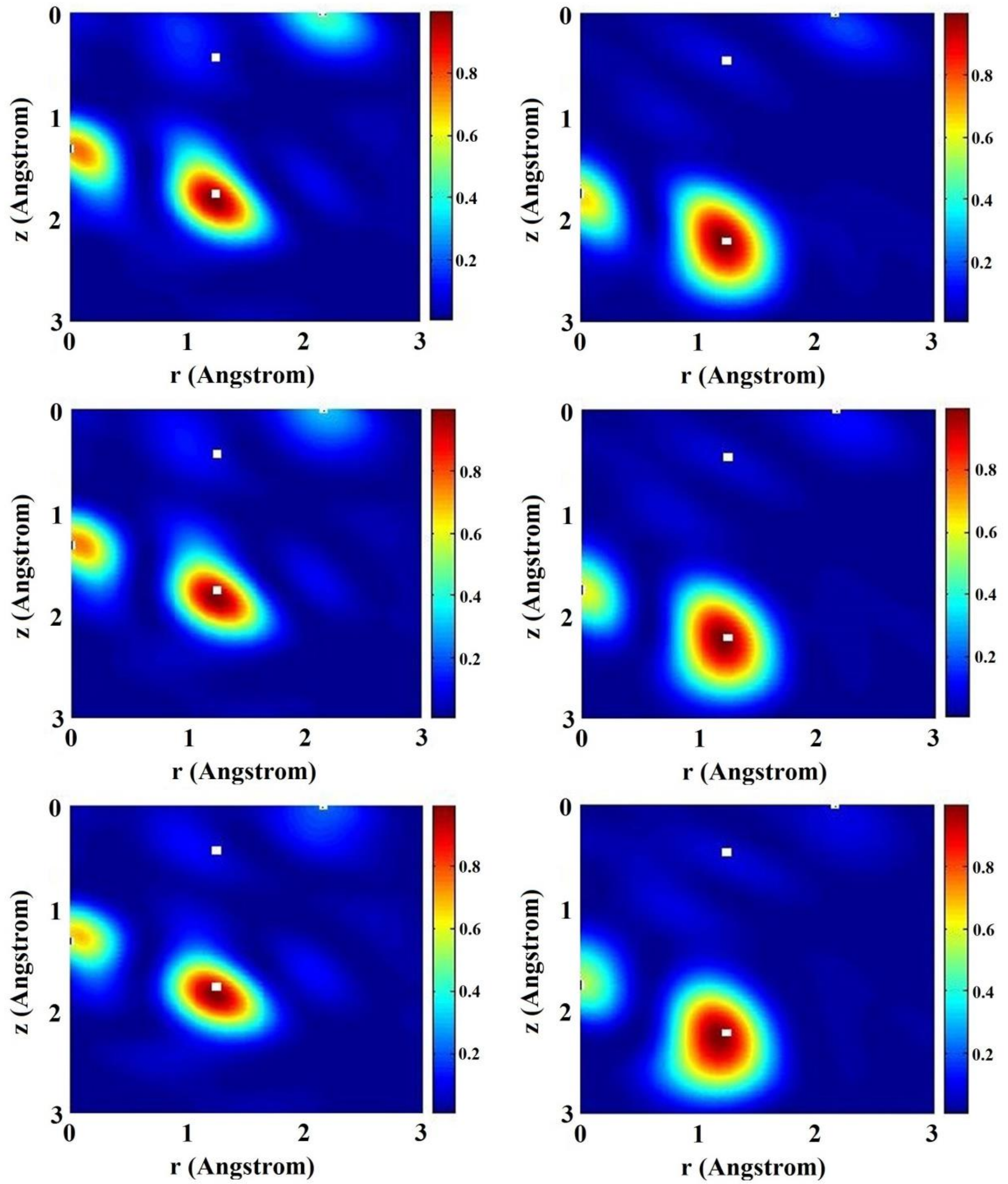


Supplementary Figure 5: 2D molecular structures transformed from the diffraction images shown in Fig. S4. The results are insensitive to the degree of molecular alignment.
